# Estimated continuous cardiac output based on pulse wave transit time in critically ill children: a report of two cases

**DOI:** 10.5935/2965-2774.20230305-en

**Published:** 2023

**Authors:** Humberto Magalhães Silva, Raisa Sanches Uzun, Isabel de Siqueira Ferraz, Marcelo Barciela Brandão, Tiago Henrique de Souza

**Affiliations:** 1 Pediatric Intensive Care Unit, Department of Pediatrics, Hospital de Clínicas, Universidade Estadual de Campinas - Campinas (SP), Brazil

**Keywords:** Cardiac output, Hemodynamic monitoring, Pulse wave analysis, Critically illness, Shock, Child

## Abstract

Cardiac output is an essential determinant of oxygen delivery, although unreliably measured on clinical examination and routine monitoring. Unfortunately, cardiac output monitoring is rarely performed in pediatric critical care medicine, with a limited availability of accurate methods for children. Herein, we report two pediatric cases in which noninvasive pulse-wave transit time-based cardiac output monitoring (esCCO, Nihon Kohden, Tokyo, Japan) was used. The esCCO system calculates cardiac output continuously by using the negative correlation between stroke volume and pulse wave transit time and requires only electrocardiogram monitoring, noninvasive blood pressure, and pulse oximetry signals. Before starting its use, esCCO should be calibrated, which can be done using patient information (gender, age, height, and body weight) or entering cardiac output values obtained by other methods. In both cases, when calibrations were performed using patient information, the agreement between esCCO and echocardiographic measurements was poor. However, after calibration with transthoracic echocardiography, the cardiac output values obtained by both methods remained similar after 2 hours and 18 hours. The results indicate that the esCCO system is suitable for use in children; however, further studies are needed to optimize its algorithm and determine its accuracy, precision, and trend in children.

## INTRODUCTION

The cardiac index and stroke volume index (SVI) are important hemodynamic parameters that require close monitoring in critically ill children. These variables are determined by dividing cardiac output and stroke volume (SV) by the patient’s body surface area. Monitoring of the cardiac index, SVI index, and systemic vascular resistance index are recommended by the main guidelines on pediatric septic shock.^(1)^ Unfortunately, continuous monitoring of the cardiac index in children is challenging and rarely performed in most pediatric intensive care units. Few cardiac output monitoring devices are applicable in children, and most have incomplete validations and/or poor accuracy.^(2)^ In addition, some methods require catheterization of arteries and deep veins, which limits their use in younger children. Finally, cardiac index estimates based on physical examination and conventional monitoring have poor correlations with objective measurements in pediatric patients with shock.^(3)^

**Figure 1:**
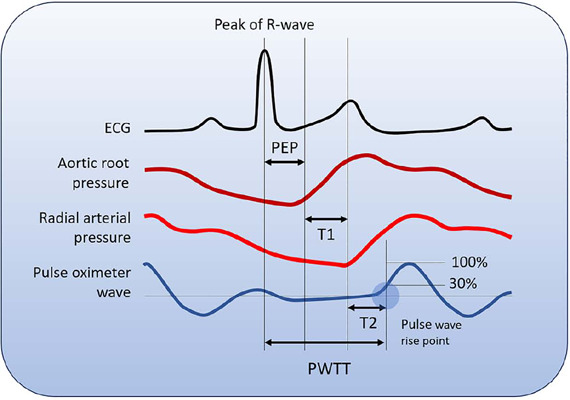
Relationship between each time-related component of pulse wave transit time

The ideal hemodynamic monitoring system must be accurate, reproducible, readily available, easy to use, operator independent, cost-effective, and safe. While fulflling these criteria is a difficult task, Nihon Kohden (Tokyo, Japan) has developed a technology with the potential to meet them: estimated continuous cardiac output (esCCO).^(4)^ This method requires only electrocardiogram monitoring, noninvasive blood pressure, and pulse oximetry signals. The esCCO algorithm calculates the cardiac index and SVI continuously by using the negative correlation between SV and pulse wave transit time (PWTT), which is the interval between the ECG R wave and a 30% increase in the pulse oximetry waveform (Figure 1). Stroke volume is directly proportional to pulse pressure and can be calculated as SV=pulse pressure x k . The constant k quantifies arterial compliance and vascular resistance and is determined at the calibration point by assigning SV and pulse pressure to the equation. The relationship between pulse pressure and PWTT can be expressed as pulse pressure=α x PWTT+β where the constant a is obtained experimentally from previous studies involving adults, while ß is determined as $\beta =\frac{VS-\kappa \times \alpha \times TTOP}{\kappa }$ . Finally, esCCO was determined using the following equation: esCCO=k x (α x PWTT+β)x heart rate . Calibration may represent a major challenge for the use of esCCO in children. Before its use, esCCO requires calibration, performed by using either patient information (gender, age, height, and body weight) or by entering cardiac output values obtained by other methods. In this report, we describe the use of esCCO in two pediatric cases in which calibration was performed using transthoracic echocardiography (TTE).

ECG - electrocardiogram; PEP - preejection period; T1 - transit time from the rising point of the aortic root pressure wave to the rising point of the radial artery pressure wave in the systolic phase; T2 - transit time from the rising point of the radial artery wave to the 30% increase in the pulse oximetry waveform in the systolic phase; PWTT - pulse wave transit time.

## CASE REPORT

### Case 1

**Figure 2:**
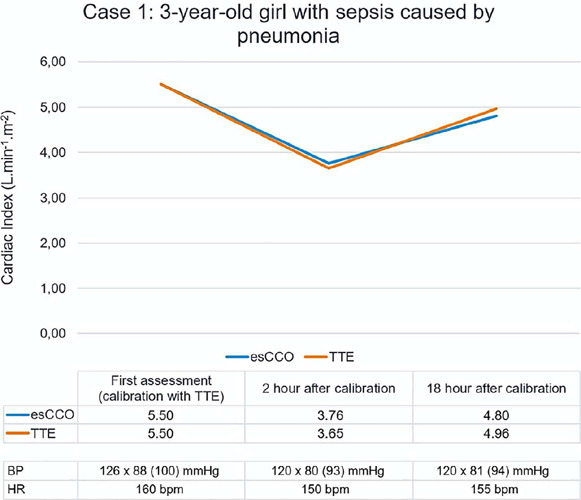
Cardiac index assessment using estimated continuous cardiac output and transthoracic echocardiography in a child admitted to the pediatric intensive care unit with sepsis

A 3-year-old girl weighing 16.8kg and height of 105cm was admitted to the pediatric intensive care unit with sepsis due to pneumonia complicated by pleural empyema. The patient was under invasive mechanical ventilation and received hemodynamic support with dobutamine at 5mcg.kg^-1^.min^-1^.m^-2^. Initially, the esCCO was calibrated with the patient’s information and showed a cardiac index of 8.87L.min^-1^.m^-2^. Simultaneously, the cardiac index obtained by TTE was 5.50L.min^-1^.m^-2^. The esCCO was then calibrated with TTE, and new echocardiography examinations were performed after 2 hours and 18 hours. The values are shown in figure 2.

esCCO - estimated continuous cardiac output; TTE - transthoracic echocardiography; BP - blood pressure; HR - heart rate.

### Case 2

**Figure 3:**
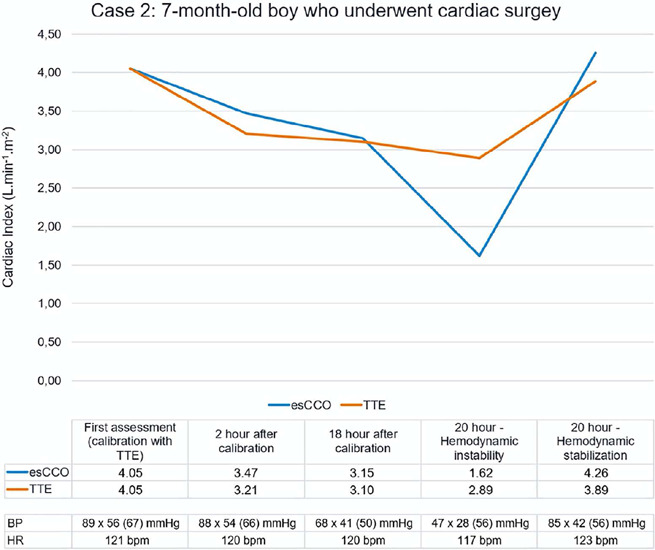
Cardiac index assessment using estimated continuous cardiac output and transthoracic echocardiography in a child who underwent cardiac surgery

A 7-month-old boy with trisomy 21 underwent surgical repair for congenital heart disease (total atrioventricular septal defect). The patient was admitted to the pediatric intensive care unit under invasive mechanical ventilation, receiving milrinone at 0.5mcg.kg^-1^.min^-1^. In the immediate postoperative period, the esCCO was calibrated with the patient’s information that showed a cardiac index of 7.05L.min^-1^.m^-2^. The esCCO was then calibrated with TTE (cardiac index of 4.05L.min^-1^.m^-2^), and new echocardiographic measurements were performed after 2 hours and 18 hours. Twenty hours after the initial assessment, the patient had hemodynamic deterioration with reduced blood pressure, prolonged capillary refill time, and weak pulses. At this time, the cardiac index measured by the esCCO was 1.62L.min^-1^.m^-2^. Hemodynamic support was optimized with fluid therapy and continuous infusion of epinephrine. After hemodynamic stabilization, esCCO showed a cardiac index of 4.26L.min^-1^.m^-2^. Echocardiography examinations were also performed to guide hemodynamic management, and the values are shown in figure 3.

esCCO - estimated continuous cardiac output; TTE - transthoracic echocardiography; BP - blood pressure; HR - heart rate.

## DISCUSSION

This report presents two common pediatric cases in which the esCCO provided valuable information to guide clinical management. In the first case, esCCO allowed the cardiac index to be closely monitored in a patient with sepsis and respiratory failure. Cardiac output is an important determinant of tissue oxygen delivery, and its monitoring is recommended to guide the resuscitation of children with septic shock.^(1,5)^ After stabilization, therapies should be directed to maintain a cardiac index greater than 3.3L.min^-1^.m^-2^ and less than 6L.min^-1^.m^-2^.^(1)^ In the second case, esCCO was able to detect a reduction in the cardiac index in the patient who underwent cardiac surgery. Patients in the postoperative period of cardiac surgery are at high risk for developing low cardiac output syndrome, which is related to increased morbidity and mortality. Episodes of hemodynamic instability need to be promptly identified and treated appropriately on time to improve outcomes.

The mainstays of hemodynamic management of shock are fluid infusions and vasoactive agents. As one of the main objectives of these therapies is to increase SV, monitoring cardiac output is essential to guide therapy. Unfortunately, clinicians have very limited ability to estimate cardiac output through physical examination and conventional monitoring, as well as to predict fluid responsiveness.^(3)^ Approximately 50% of fluid infusions result in increases in SV.^(6)^ When advanced hemodynamic monitoring is not available, systemic blood pressure is often used as a surrogate for cardiac output. However, this practice can lead to inappropriate clinical decisions. Several studies have shown that changes in arterial pressure are not correlated with changes in cardiac index after fluid infusion in children with septic shock.^(7,8)^

To the best of our knowledge, this is the first description of cardiac output monitoring using pulse wave transit time in young critically ill children. The first and only report on the use of this method in children was published in 2013.^(9)^ Terada et al.^(9)^ evaluated the agreement between esCCO and pulse dye densitometry for cardiac output measurement in 10 adults and 7 children undergoing kidney transplant surgery. The mean age and weight of the children were 9.4 years and 25.5kg, respectively. After initial calibration with pulse dye densitometry, cardiac output was assessed twice in each patient by both methods, before declamping the artery and at the end of surgery. The correlation coefficient between esCCO and pulse dye densitometry was 0.904 for children and 0.756 for adults. Additionally, there were 95% better limits of agreement between the methods and a lower percentage error were observed in children than in adults (35.7% *versus* 42.7%, respectively). The authors concluded that esCCO is useful for pediatric patients in clinical settings, although they recognize that its utility is limited if invasive measurement is required for calibration.

Calibration may be the most important limitation for the use of esCCO. Although calibration with patient information is useful in adults, it does not appear to be valuable in children. Further studies are needed to improve the pediatric population’s esCCO algorithm or to determine the time interval in which recalibration with another method is necessary. In the cases reported, when calibrations were performed using patient information, the agreement between esCCO and TTE measurements was poor. However, after calibration with the TTE, the cardiac index values obtained by both methods remained similar after 2 hours and 18 hours. Although the reference method was not thermodilution, the gold standard technique, pediatric Doppler cardiac output measurements have accuracy, precision, and acceptable repeatability. Furthermore, thermodilution is not as accurate as previously thought, especially as cardiac output increases.^(10)^ Therefore, further research should focus not only on accuracy and precision but also on the ability to follow changes in cardiac output accurately.

## CONCLUSION

The reported cases showed that continuous monitoring of cardiac output is valuable in providing adequate hemodynamic management; therefore, routine monitoring of cardiac output should be encouraged in pediatric intensive care units. Estimated continuous cardiac output has interesting features that make this method potentially suitable for use in children. Further studies are needed to determine its accuracy, precision, and trend in the pediatric population.
